# Oral Human Papillomavirus: a multisite infection

**DOI:** 10.4317/medoral.23462

**Published:** 2020-03-06

**Authors:** María Inés Criscuolo, Rosana A. Morelatto, Paola A. Belardinelli, Jessica Mosmann Mosmann, Cecilia Cuffini, Silvia A. López de Blanc

**Affiliations:** 1 School of Dentistry, Universidad Nacional de Córdoba. Córdoba, Argentina; 2 Virology Institute. School of Medicine.Universidad Nacional de Córdoba. Córdoba, Argentina

## Abstract

**Background:**

The Human Papillomavirus (HPV) has different strategies for persist in the cells. This characteristic has led us to consider the presence of the virus in tissues of the oral cavity that had no clinical signs of infection. The aim of this study was to detect the presence of DNA-HPV at multiple sites of the oral cavity.

**Material and Methods:**

A case-control study was designed: Oral Squamous Carcinoma Group (OSCG), healthy n=72 and Control Group (CG), n=72, healthy volunteers paired by sex and age with OSCG. Four samples were taken from OSCG: saliva, biopsy, brush scraping of lesion and contralateral healthy side. In CG a saliva sample and a scratch of the posterior border of tongue were collected. HPV was detected by PCR using Bioneer Accuprep genomic DNA Extraction kit, and consensus primers MY09 and MY11. Chi square test was applied.

**Results:**

432 samples were obtained from 144 individuals. DNA-HPV was detected in 30 (42%) of OSCG subjects and 3(4%) of CG. Two or more positive samples were obtained in 67% of the OSCG, 67% in saliva and 60% in biopsy; in CG 100% of the individuals were positive in the two samples.

**Conclusions:**

HPV is frequently present in oral cavity as a multifocal infection, even without the presence of clinical lesions.

** Key words:**HPV, Oral cavity, Saliva, Oral cancer.

## Introduction

The first cells to become in contact with the human papillomavirus (HPV) in the oral cavity, as in the genital mucosa, are undifferentiated cells of the basal layer of the epithelium. This contact implies the previous existence of ulceration or micro-wounds on the epithelial surface that allows its entry (1-3). Viral DNA and RNA transcriptions occurs in the basal layer, whereas viral replication occurs in the most differentiated layers of the epithelium (4,5). This association between viral replication and progressive differentiation would involve a strategy to persist in a host with high cell proliferation (6). In addition, the virus can evade the innate immune system, delaying the adaptive immune response. Infected cells are pushed out towards the epithelial surface, away from the circulating immune system, and therefore no viremia occurs. In conclusion, the lack of cell death and inflammatory response makes HPV a “successful microorganism” for infection (7). This characteristic of the virus has led us to consider the possible presence of HPV in saliva or samples of other tissues of the oral cavity that had no clinical signs of infection and to think whether the presence of the virus could occur silently or latently in multiple sites of the oral cavity simultaneously. We also consider, as other authors, that areas of constant rubbing in the mouth, such as the lateral border of the tongue, could be the first site of exposure to the virus (8).

We do not find in the current literature at our disposal, studies on the presence of simultaneous HPV infection in different tissues of the oral mucosa. Nevertheless, some authors reported the presence of HPV in different sites of the anogenital tract without clinical symptoms of infection. These studies report cases of simultaneous infection of the oral and genital mucosa (9-11). Studies on the prevalence of oral HPV infection in healthy individuals have revealed a high variability in different populations, with values ranging between 0.2% and 20.7% (12). Tables shows prevalence of oral HPV infection in healthy individuals of different populations.

The objective of this work was to detect the presence of DNA-HPV in multiple sites of the oral cavity.

## Material and Methods

A case-control study was designed and conducted between 2010 to 2017, which was approved by the Institutional Committee of Ethics in Health Research from the School of Dentistry, Universidad Nacional de Córdoba (CIEISN°6/2010). All the subjects signed an informed consent.

- Study groups

Two groups were considered:

a- Oral Squamous Carcinoma Group (OSCG): 72 patients with diagnosis of oral squamous cells carcinoma; b- Control Group (CG):72 clinically healthy at the oral cavity, which means, with any oral clinical lesions at the time of this study, paired by sex and age (+/- 5) with the OSCG. This group was relieved from a population study titled “Survey of oral health parameters in the adult population from Córdoba city 2014–15” done in the city of Córdoba, Argentina. A multistage cluster sampling was conducted, with random selection of census tracts, probability proportional to size, blocks, households, and persons; the same number of individuals per tract was selected. The included subjects were asked to attend a health center in the area. Pregnant women and subjects under age 18 were excluded. The results were informed by phone or via mail to patients and healthy controls and they were also clinically controlled.

- Data collection

Clinical history of all patients was recorded, including personal data, personal and hereditary records, risk factors and oral health state. Subjects were inspected by personnel trained for clinical diagnosis. OSCG were subjected to biopsy to obtain diagnostic certainty. The data on tobacco consumption was made according the number of pack year smoked; a subject that smoked fifteen or more pack years in twenty years was considered a smoker (13). One alcohol unit a day (one drink) was considered as regular alcohol consumption (14).

- Sample collection

Four samples per patient were obtained from the OSCG: 1-unstimulated whole saliva collected in sterile tube; 2-cytobrush of lesion; specimens were placed in sterile tube containing 2 ml phosphate buffer solution with antibiotic and antifungal agents; 3-cytobrush of healthy contralateral mucosa to the lesion site, as in 2; and 4- biopsy of lesion; samples were placed in sterile tube in fresh conditions. All samples were preserved at 4°C until DNA extraction. The CG group was subjected to saliva sampling and cytological analysis of the posterior border of the tongue, using the same technique as in OSCG. In this group, we chose this area for being one of the most exposed to micro-wounds that would facilitate the entry of the virus.

- Sample processing

DNA-HPV was detected via PCR using Bioneer AccuPrep genomic DNA Extraction kit, and consensus primers MY09 and MY11. These primers amplified a fragment of approximately 450 base pairs corresponding to the highly conserved L1 region of the virus genome. The method involved a 50 μl reaction mix containing 10 μl DNA, 1.5 mM MgCl2, 200 μM of each dNTP (dATP, dCTP, dGTP and dTTP), 10 pmoles of each oligonucleotide primer and 1 U Taq DNA polymerase (Promega Corporation, Madison, USA). The temperature profile used for amplification constituted an initial denaturation at 94°C for 3 min followed by 35 cycles with denaturation at 94°C for 1 min, annealing at 55°C for 1 min and extension at 72°C for 1 min which was extended for 5 min in the final cycle. Typing was performed by means of the analysis of the amplified product using the enzyme restriction fragment length polymorphism (RFLP) with the following restriction enzymes: Bam H1, Dde I, Hae III, Hinf I, Pst I, Rsa I, and Sau3 AI. A mixed of PCR product and buffer was prepared to each enzime separately for subsequent addition of individual restriction enzymes. The mix included 6.5 µl PCR product, 2 µl buffer, 1.5 µl restriction enzyme, followed by incubation 40ºC for 60 min. The result was a characteristic banding pattern visualized with UV light (15). Subjects with at least one positive sample were considered HPV+, this means an HPV infection of the sample.

- Statistical analysis

Data were expressed as relative frequencies; data association was analyzed using the chi square test, and OR was used to reveal the intensity of the association.

## Results

Two study groups were formed with 144 subjects, corresponding 72 to each group. The total of samples obtained from both was 432. Table 1 describes the general characteristics of both groups or of each of the two groups.

- Sample analysis: DNA-HPV was detected in 30 (42%) patients of OSCG and 3 (4%) in subjects of CG. The analysis and comparison of the results of OSCG with controls showed a highly significant difference, with an OR of 16.43 (CI: 5.10-52.94), *p*<0.0001 (Table 2).

- DNA-HPV at multiple sites: 67% of the HPV+ patients of OSCG had two or more positive samples. In the case of CG, 100% of the cases were positive in both samples Table 3 describes the distribution of positive samples in both groups. Four positive samples prevail in OSCG, where the most frequent positive sample was in saliva (67%), followed by biopsy (60%).

**Table 1:**
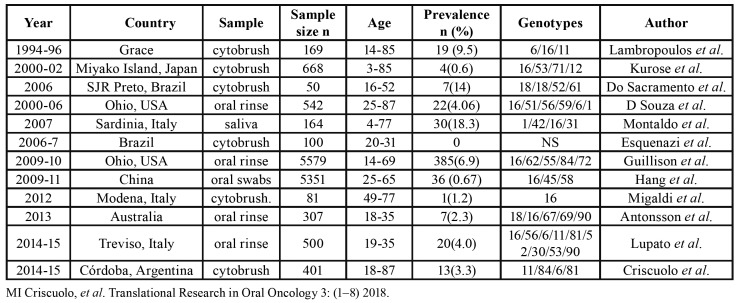
Prevalence of oral HPV infection in healthy individuals.

**Table 2:**
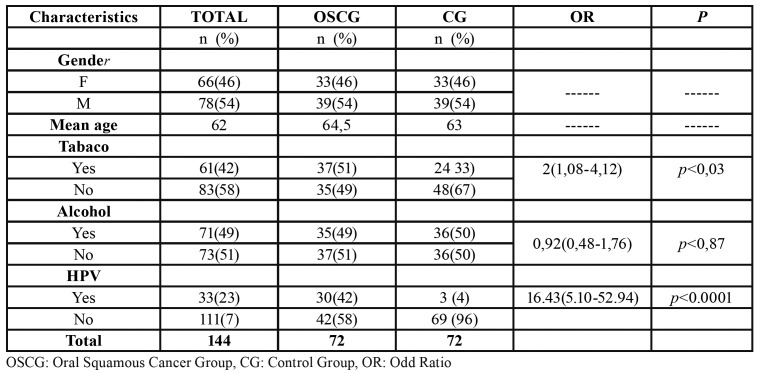
General characteristics of the study groups.

**Table 3:**
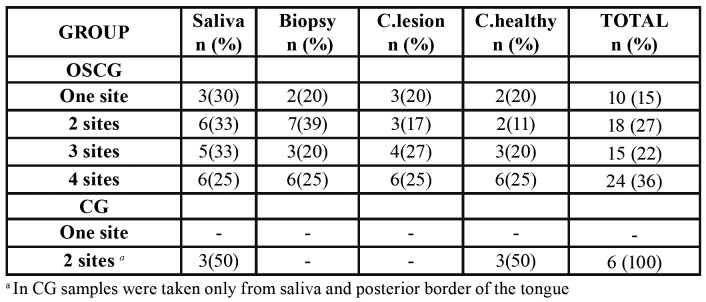
Distribution of HPV in different sites.

When positive patients in two or more sites in the OSCG were analyzed and was related with the tumors site, we observed that the 65% were located at the border of the tongue and oropharynx.

- Genotypes: The genotypes were 16/ 6/ 11/ 31/ 33/ 45/ 52/ 53/ 69, with 16/ 6/ 11 being the most frequent. In nine samples of the OSCG it was not possible to identify the genotype. In the OSCG, 12 patients (40%) had high-risk genotypes: 16/ 52/ 45/ 31/ 69 and 33, with 50% of these being HPV 16. Of the remaining positive types, 9 patients (30%) were of low risk (6/11); one of these patients had combination of genotypes 6 and 11, and of the remaining nine patients (30%) were not genotyped. In CG all genotypes were of low risk (11/84).

## Discussion

The results of the present work show the prevalence of positive samples in more than one site of the oral cavity in DNA-HPV positive subjects of the groups studied. We also observed that in healthy mucosa the virus was present despite the lack of clinical signs of infection. In fact, 100% of controls and 43% of patients with oral cancer were positive in healthy mucosa.

Some authors assume that HPV could present as a multifocal infection in different mucous membranes (16-19). Our results effectively confirm that oral DNA-HPV appears as a simultaneous infection in different sites of the mouth.

In spite of not finding in the current literature, studies like ours, we found some authors who had studied the virus in different conditions such as HIV positive patients or groups at risk for diseases such as sexually transmitted diseases. Authors like Ciccarese *et al*. studied the HPV in a risk group of sexually transmitted diseases, and evaluated the presence of the virus at different sites with no clinical signs of infection. They took samples from the oral, genital and anal mucosa. The highest values were recorded in genital samples, and 37% of HPV positive values were obtained in the oral cavity. This result is similar to the values found in the samples taken in the present work in the carcinoma group. It should be noted that although they obtained similar percentages in both sexes, 51% of HPV positive males in the oral cavity had simultaneous genital infection, resulting in a significant association, with an OR of 3.63 (CI 1.37-9.58) (9). On the other hand, Ucciferri *et al*. studied HPV in 90 males, 45 HIV+ and 45 HIV-, in oral, anal, urethral mucosa and coronal sulcus samples. The results showed that 59% of the subjects had infection at multiple sites, 34% at two sites and 22% at three sites. It is important to note that in oral mucosa HPV positive patients did not exhibit different values between HIV positive and negative subjects (10). In a similar study (20) where studied HPV at oral, genital and anal mucosa in HIV positive and negative males, observed that the virus was detected in the three samples simultaneously in 14% and 10% respectively. In Peru, Blas *et al*. studied the detection of HPV at multiple sites in men who had sex with men, HIV positives and negatives; they obtained samples from oral, anal, and genital mucosa and blood. Although the report does not provide the percentage of HPV positive cases at several sites simultaneously, at the oral cavity level the values were similar in both groups, with 8.5% in HIV positive subjects and 9.9% in HIV negative subjects. The highest HPV percentage recorded was in anal mucosa of HIV positive patients; high risk genotypes were the most prevalent (86%) in this group and at this site, and HPV 16 was the most frequent. In oral mucosa, HIV positive cases had a higher percentage of high-risk genotypes than HIV negative cases (21,22). In our study, the most frequent genotypes were 16/6/11 and the oral cancer group was the one with highest percentage of high-risk HPV and, as in the previously mentioned study, HPV 16 was the most frequent genotype. In the current clinical cases, we do not find HPV 18, a high-risk genotype that is frequently found in the literature (23). Table 4 compares the prevalence of HPV in OSCC patients of different studies and the highest frequency of HR-HPV in these groups (24).

**Table 4:**
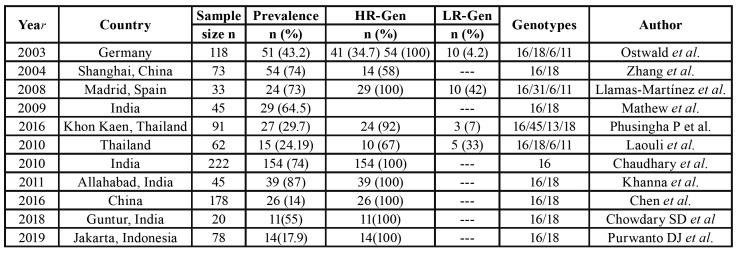
Prevalence of HPV in OSCC patients.

We believe important that these results of virus detection and the expression of predominant genotypes in both sexes are useful to support educational campaigns and the need to apply HPV preventive vaccines. Although the World Health Organization recommends the inclusion of males in HPV vaccination strategies, only in some countries like Argentina these are applied preventively since 2017, in both sexes (25).

Regarding our findings of a high positive DNA-HPV percentage in saliva, similar to that in tissue subjected to biopsy, several authors have discussed the utility of detecting the virus in this fluid. Zhao *et al*. analyzed HPV 16 in saliva of patients with head and neck carcinoma and compared it with a healthy control group; they found significant differences between groups (26). In our results, saliva samples of all the HPV positive cases of the healthy volunteer group were positive, despite the lack of lesions suggestive of DNA-HPV infection.

## Conclusions

Oral cavity HPV often occurs as a multifocal infection, even without the presence of clinical lesions. The current vaccination scheme in Argentina prevents against the genotypes circulating in our medium. Future studies are necessary to assess the clinical and epidemiological implications of the virus presence in saliva.
